# Structural biology in the time of COVID-19: perspectives on methods and milestones

**DOI:** 10.1107/S2052252521003948

**Published:** 2021-04-23

**Authors:** Miranda L. Lynch, Edward H. Snell, Sarah E. J. Bowman

**Affiliations:** a Hauptman–Woodward Medical Research Institute, 700 Ellicott Street, Buffalo, NY 14203, USA; b Department of Materials Design and Innovation, The State University of New York at Buffalo, Buffalo, NY 14203, USA; c Department of Biochemistry, Jacobs School of Medicine and Biomedical Sciences at The State University of New York at Buffalo, Buffalo, NY 14023, USA

**Keywords:** structural biology, SARS-CoV-2, X-ray crystallography, cryoelectron microscopy, COVID-19

## Abstract

The global COVID-19 pandemic caused by severe acute respiratory syndrome coronavirus 2 (SARS-CoV-2) has wreaked unprecedented havoc on global society, in terms of a huge loss of life and burden of morbidity, economic upheaval and social disruption. Yet the sheer magnitude and uniqueness of this event has also spawned a massive mobilization of effort in the scientific community to investigate the virus, to develop therapeutics and vaccines, and to understand the public health impacts. Structural biology has been at the center of these efforts, and so it is advantageous to take an opportunity to reflect on the status of structural science vis-à-vis its role in the fight against COVID-19, to register the unprecedented response and to contemplate the role of structural biology in addressing future outbreak threats. As the one-year anniversary of the World Health Organization declaration that COVID-19 is a pandemic has just passed, over 1000 structures of SARS-CoV-2 biomolecules have been deposited in the Worldwide Protein Data Bank (PDB). It is rare to obtain a snapshot of such intense effort in the structural biology arena and is of special interest as the 50th anniversary of the PDB is celebrated in 2021. It is additionally timely as it overlaps with a period that has been termed the ‘resolution revolution’ in cryoelectron microscopy (CryoEM). CryoEM has recently become capable of producing biomolecular structures at similar resolutions to those traditionally associated with macromolecular X-ray crystallo­graphy. Examining SARS-CoV-2 protein structures that have been deposited in the PDB since the virus was first identified allows a unique window into the power of structural biology and a snapshot of the advantages of the different techniques available, as well as insight into the complementarity of the structural methods.

## Abstract 2

The one-year anniversary of the declaration of the outbreak of SARS-CoV-2 as a pandemic, combined with the 50th anniversary of the PDB, provides a unique occasion to observe the role played by structural methods in the fight against COVID-19. A revealing portrait of the many contributions of the structural biology community is presented.

## 1. Structural methods   

**Figure 1:**
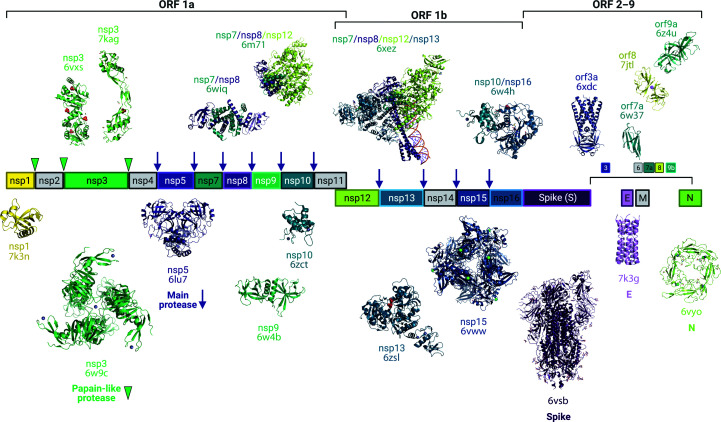
Map of SARS-CoV-2 protein structures across the open reading frames of the viral genome. Each structure depicted is the first deposited structure of that particular SARS-CoV-2 protein in the PDB [PDB entries 6vxs (Michalska *et al.*, 2020 ▸), 7kag (Center for Structural Genomics of Infectious Diseases, unpublished work), 6wiq (Center for Structural Genomics of Infectious Diseases, unpublished work), 6m71 (Gao *et al.*, 2020 ▸), 6xez (Chen *et al.*, 2020 ▸), 6w4h (Rosas-Lemus *et al.*, 2020 ▸), 6xdc (Kern *et al.*, 2021 ▸), 7jtl (Flower *et al.*, 2021 ▸), 6w37 (Center for Structural Genomics of Infectious Diseases, unpublished work), 6z4u (S. D. Weeks, S. De Graef & A, Munawar, unpublished work), 7k3n (Semper *et al.*, 2021 ▸), 6w9c (Center for Structural Genomics of Infectious Diseases, unpublished work), 6lu7 (Jin *et al.*, 2020 ▸), 6w4b (Center for Structural Genomics of Infectious Diseases, unpublished work), 6zct (Rogstam *et al.*, 2020 ▸), 6zsl (J. A. Newman, Y. Yosaatmadja, A., Douangamath, C. H. Arrowsmith, F. von Delft, A. Edwards, C. Bountra & O. Gileadi, unpublished work), 6vww (Kim *et al.*, 2020 ▸), 6vsb (Wrapp *et al.*, 2020 ▸), 7k3g (Mandala *et al.*, 2020 ▸) and 6vyo (Center for Structural Genomics of Infectious Diseases, unpublished work)]. The cleavage sites of the proteases are shown by green triangles (papain-like protease) and purple arrows (main protease).

In the weeks after the first report of the cluster of pneumonia cases caused by the novel coronavirus, the first SARS-CoV-2 genome sequence was made publicly available (Wu *et al.*, 2020 ▸). Within hours, structural biologists worldwide had pivoted to focus on solving the structures of the biomolecules that make up the virus. In a matter of just two weeks, the first three-dimensional structure of one of the critical viral proteins, the main protease, was solved using macromolecular X-ray crystallography (MX) and deposited in the PDB (PDB entry 6lu7), and was released just over a week later (Jin *et al.*, 2020 ▸). The first CryoEM structure, of the spike protein, was similarly deposited within weeks of the genome release (PDB entry 6vsb; Wrapp *et al.*, 2020 ▸). Structural biology has played crucial roles in impacting our understanding of how the viral mechanisms work, how different viral proteins interact with human host proteins and how ligands bind to the viral proteins (Baker, 2020 ▸; Subramaniam, 2020 ▸). Structural biologists have investigated a substantial number of the proteins encoded by the viral genome, which have provided essential details about how the virus functions (Fig. 1 ▸).

The spike protein drives viral entry into host cells and is therefore the main vaccine-development target. It serves as an example of the structural approach, with studies revealing the spike protein in a number of conformations, in complex with the human ACE2 receptor and in complex with human antibodies (Barnes *et al.*, 2020 ▸; Shang *et al.*, 2020 ▸; Walls *et al.*, 2020 ▸; Wrapp *et al.*, 2020 ▸; Yuan *et al.*, 2020 ▸). Vaccine development has benefitted from 3D structures of the spike protein (Vogel *et al.*, 2020 ▸). Once the virus is inside the host cell, it is able to hijack the host cellular machinery directly to begin translation of viral RNA. The largest viral gene (orf1ab) comprises two overlapping reading frames that encode two polyproteins that are cleaved into 16 nonstructural proteins (nsp1–nsp16; Gordon *et al.*, 2020 ▸; V’kovski *et al.*, 2020 ▸). Two cysteine proteases, the papain-like protease encoded by a portion of nsp3 and the main protease encoded by nsp5, function to process these precursor proteins into all of the constituent nsp proteins required for directing SARS-CoV-2 replication, transcription and viral assembly. These proteins, including the two proteases, RNA polymerase, helicase and endonuclease, are especially attractive targets for the development of thera­peutics (Douangamath *et al.*, 2020 ▸; Gao *et al.*, 2020 ▸; Gordon *et al.*, 2020 ▸; Jin *et al.*, 2020 ▸; Kneller, Phillips, O’Neill *et al.*, 2020 ▸; Newman *et al.*, 2021 ▸; Shin *et al.*, 2020 ▸; Schuller *et al.*, 2021 ▸; Yin *et al.*, 2020 ▸; Zhang, Lin *et al.*, 2020 ▸). Significant progress has been made in solving the structures of the nonstructural proteins, including a substantial number of structures with potential inhibitors and fragments bound (Douangamath *et al.*, 2020 ▸; Newman *et al.*, 2021 ▸; Schuller *et al.*, 2021 ▸).

The speed with which these structural investigations have occurred has been breathtaking. Simultaneously, the availability of these data has benefitted from the long history of making structural data and metadata accessible via the PDB, the worldwide data repository for macromolecular structures (Berman *et al.*, 2003 ▸). As structural biologists, we have an extensive history of open access and resource sharing; these tendencies and the platforms already established by the community for data sharing have encouraged even more rapid dissemination of structural details during the pandemic. Even in our resource-sharing-rich community, the level and swiftness of data sharing over the last year have been unprecedented. There have been several reviews of SARS-CoV-2 protein structures that have been solved and the insights that the structural details provide into viral mechanisms (Bárcena *et al.*, 2021 ▸; Mariano *et al.*, 2020 ▸; Wang *et al.*, 2020 ▸). While the number of structures of SARS-CoV-2 biomolecules deposited in the PDB increases weekly, the trends that we report here have remained consistent since we began tracking these details in early 2021. The goal of this perspective piece is to provide a snapshot of this instant in time, a year into the COVID-19 pandemic, as a window into the structural biology techniques being used and what these outcomes might mean for the future of structural methods.

### 1.1. Experimental approaches for SAS-CoV-2 biomolecules   

The first SARS-CoV-2 structure deposited in the PDB (PDB entry 6lu7; Jin *et al.*, 2020 ▸), which was deposited on 26 January 2020, was of the main protease, a major therapeutics development target, and was swiftly followed by 23 more SARS-CoV-2 protein structures in February 2020. Since January 2020, a total of 9243 structures have been deposited and released in the PDB; of these, an astonishing 1038 structures have been of SARS-CoV-2 proteins. In other words, ∼11% of all structures deposited in the PDB between 1 January 2020 and 10 March 2021 have been SARS-CoV-2 structures. These numbers paint a compelling picture of the dramatic pivot towards studying SARS-CoV-2 that the structural biology community has made in response to the COVID-19 pandemic. Of additional note in these numbers is the steep decline in the total number of structures that have been deposited during the course of the pandemic. Querying similar time frames over the prior two-year period yields total counts of deposited structures of 14 706 from 1 January 2019 to 11 March 2020 and 13 796 from 1 January 2018 to 13 March 2019. While 9243 structures represent a decrease of over one-third of the expected structure depositions in this time period based on recent history, it also reflects some level of productivity of structures during the pandemic. These structural depositions could comprise a combination of data collected previously that could be worked on during lockdown periods and new data enabled by remote data-collection capabilities. The long-term impacts of the worldwide lockdowns on scientific research remain to be seen in their entirety, but even at this point it is clear that there will be enduring consequences.

**Figure 2:**
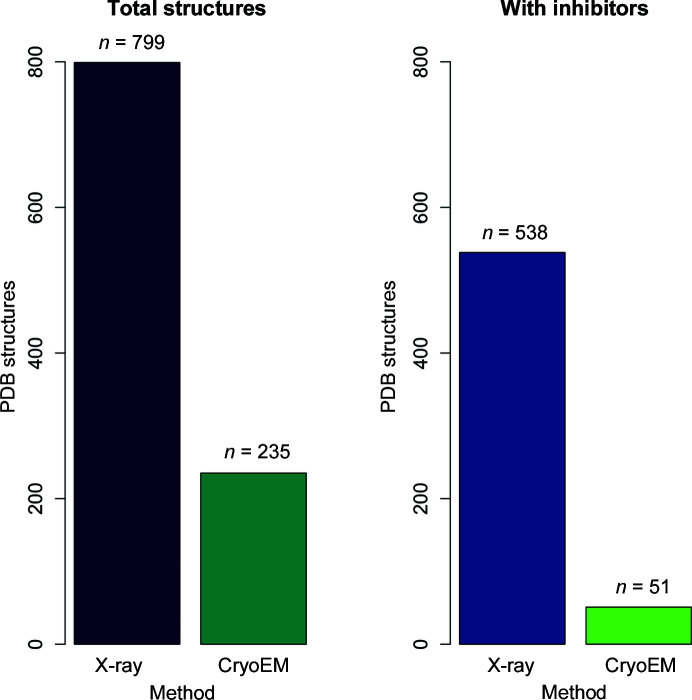
Bar plots of deposited and released structures of SARS-CoV-2 samples. Left: of the 1038 SARS-CoV-2 structures in the PDB as of 10 March 2021, 1034 were solved using MX and CryoEM methods, with a ratio of ∼77% to ∼23%. Right: when considering the subset of structures with inhibitors or fragments bound, the ratio is ∼91% MX and ∼9% CryoEM.

The pandemic has given us an unprecedented opportunity to interrogate the contributions of different structural biology methods. This snapshot shows that a year into the pandemic, ∼77% of the structural information about SARS-CoV-2 comes from MX and ∼23% from CryoEM (Fig. 2 ▸). However, ∼91% of our knowledge of how ligands interact with viral target proteins comes from MX. In some ways, these outcomes may reflect the availability of established approaches and pipelines. MX methods have benefitted from well established procedures, including high-throughput crystallization, high-throughput data-collection methods and high-throughput ligand screening. Structural biology plays an important role in structure-based drug design, and the pressing need for the development of vaccines and therapeutics against SARS-CoV-2 enabled the immediate application and extension of these high-throughput methods. Indeed, work by the XChem Team at Diamond Light Source and by the Coronavirus Research Group at UCSF has exploited recently developed fragment-screening MX methods that have been instrumental in the identification of potential chemicals and fragments that bind the main protease, helicase, endonuclease and macrodomain of nsp3, even for fragments that may not be bound as tightly at the active site, may be bound at possible allosteric sites or are found with lower occupancies (Douangamath *et al.*, 2020 ▸; Newman *et al.*, 2021 ▸; Schuller *et al.*, 2021 ▸). These high-throughput fragment-screening efforts have resulted in at least 428 SARS-CoV-2 structures that have been deposited in the PDB with a wide variety of bound fragments.

**Figure 3:**
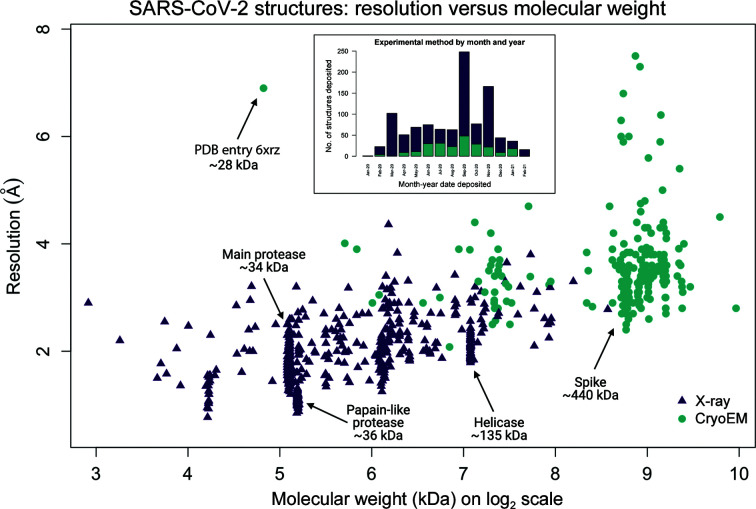
SARS-CoV-2 structures deposited in the PDB. The scatter plot displays resolution versus molecular weight for the deposited structures solved using MX (purple triangles) or CryoEM (teal circles) methods. Proteins with a number of structures deposited are noted; for instance, there are 280 structures of the main protease, over 200 of which are bound to inhibitors or fragments. The outlier RNA CryoEM structure (PDB entry 6xrz) is also noted. Inset: bar plots showing the number of structures deposited and the experimental method used by month and year from January 2020 to February 2021.

To further probe the contributions of the structural biology methods MX and CryoEM in the fight against COVID-19, we examined details regarding the deposition date, resolution and molecular weight of SARS-CoV-2 structures (Fig. 3 ▸). We anticipated that the initial structural approaches would be biased towards CryoEM, in part because of the well known difficulties in finding crystallization conditions for protein targets. We were surprised to discover that the structures deposited in the first few months of the pandemic were nearly all solved using MX methods (Fig. 3 ▸, inset). In general, the lower molecular weight SARS-CoV-2 biomolecules have primarily been solved using MX techniques, while those at higher molecular weight have primarily been solved using CryoEM methods. The smallest structure solved via CryoEM is an outlier as a 28.3 kDa RNA structure, although its resolution is only 6.9 Å (PDB entry 6xrz; Zhang, Pintilie *et al.*, 2020 ▸). Except for this RNA structure, all structures of SARS-CoV-2 samples below 50 kDa have exclusively been solved by MX. Similarly, all structures and complexes above 380 kDa have exclusively been solved by CryoEM. There is a range of biomolecules with molecular-weight values between ∼100 and ∼220 kDa that are accessible by both methods. This analysis emphasizes the necessity of bringing the entire arsenal of structural techniques to bear and highlights the capabilities and complementarity of the MX and CryoEM methods.

### 1.2. Computational approaches for SAS-CoV-2 proteins   

The physical models of the various SARS-CoV-2 proteins derived by experimental methods have been critical for unlocking the atomic-level details that have underpinned drug discovery and vaccine development. The SARS-CoV-2 pandemic has also shone a light on the many diverse contributions of *in silico* structural biology work (Mulholland & Amaro, 2020 ▸). Initial structure-prediction work using *C-I-TASSER* (Zhang, Zheng *et al.*, 2020 ▸; Zhang, 2020 ▸) and *AlphaFold* from the DeepMind consortium (Jumper *et al.*, 2020 ▸; Senior *et al.*, 2020 ▸) was able to rapidly leverage the released genomes, determine protein sequences and create computational models that appeared in early February and March 2020. Molecular-dynamics simulations have massively extended the utility of experimental structural data, providing novel insights via the rapid analysis of *in silico* mutagenesis for many of the key viral proteins (Casalino, Dommer *et al.*, 2020 ▸; Rynkiewicz *et al.*, 2021 ▸; Sheik Amamuddy *et al.*, 2020 ▸) and the ability to model glycosylation of the spike protein (Casalino, Gaieb *et al.*, 2020 ▸; Woo *et al.*, 2020 ▸). Computational approaches have also helped to probe potential host–pathogen protein interactions (HPIs), contributing network-based and machine-learning-based assessments of putative interactions leveraging multiple HPI databases (Dey *et al.*, 2020 ▸; Messina *et al.*, 2020 ▸). One ambitious project, *Folding@Home*, has even employed crowdsourcing to access exascale computing resources for multiple SARS-CoV-2 simulation projects (Achdout *et al.*, 2020 ▸; Zimmerman & Bowman, 2021 ▸). The synergy between computational models and experimental methods, especially in the areas of protein dynamics, glycosyl­ation and protein-interaction studies, has propelled both approaches forward.

## 2. Materials and methods   

The PDB structures in Fig. 1 ▸ were generated with *PyMOL* (version 2.4.0; Schrödinger) curated by SBGrid (Morin *et al.*, 2013 ▸). Fig. 1 ▸ was created using the *BioRender* software. All PDB data in Figs. 2 ▸ and 3 ▸ were extracted from the PDB on 10 March 2021 (Burley *et al.*, 2020 ▸). These data include a total of 1038 SARS-CoV-2 structures that have been deposited and released. Four structures were solved using NMR spectroscopy and are not included in Figs. 2 ▸ and 3 ▸. The two structures solved by joint neutron and X-ray diffraction methods [PDB entries 7jun (Kneller, Phillips, O’Neill *et al.*, 2020 ▸) and 7lb7 (Kneller *et al.*, 2021 ▸)] were included in the MX totals. We note that the scatter plot in Fig. 3 ▸ does not show structures at >8 Å resolution or with molecular weight >1000 kDa (a total of 16 structures are not shown in the scatter plot). Bar plots and scatter plots were generated using the *R* statistical software (R Core Team, 2020 ▸) and the* viridisLite* palette package (Garnier, 2018 ▸).

## 3. Discussion: rumors of the death of X-ray crystallography have been greatly exaggerated   

The structural biology community has come together in a tour de force of collegiality and cooperation to rapidly address the COVID-19 pandemic. The past year has seen an impressive leveraging of remote capabilities from crystallization screening supported by our laboratory at Hauptman–Woodward Medical Research Institute (Iketani *et al.*, 2021 ▸; Kneller, Phillips, O’Neill *et al.*, 2020 ▸; Kneller, Phillips, Weiss *et al.*, 2020 ▸; Ye *et al.*, 2020 ▸) to the remarkable fragment screening and data collection that has occurred with limited staffing at synchrotron beamlines and CryoEM facilities around the world. The availability of open-source materials has had a critical impact on our ability to solve structures of SARS-CoV-2 biomolecules, from the initial distribution of the genome and data hosting on GISAID to structural depositions in the PDB. Indeed, structures were often being deposited and released in the PDB before even preprint manuscripts were released. We have a more refined picture of the individual capabilities and complementarity of our structure-determination methods. The pandemic has revealed that even in the CryoEM resolution revolution (Kühlbrandt, 2014 ▸), where it has become capable of producing biomolecular structures at similar resolutions to those traditionally associated with MX (Nakane *et al.*, 2020 ▸; Zhang, Pintilie *et al.*, 2020 ▸), crystallo­graphy excels in the ability to yield high-resolution structures of low-molecular-weight targets and to rapidly screen fragments and inhibitors for structural knowledge. The specific contributions of MX and CryoEM to structures of SARS-CoV-2 biomolecules emphasize how the methods complement each other and have been required in the fight against COVID-19. The high level of information sharing has also empowered the deployment of several online resources, including collaborative efforts to re-refine initial structural models (Brzezinski *et al.*, 2021 ▸; Croll *et al.*, 2020 ▸). Computational approaches have enabled the study of glycosylation and emerging variants using MD simulations. Integration of all of these efforts, and the synergy between experimental structural work and computational models, has led to fundamental advancements in pinning down the mechanistic details of the SARS-CoV-2 virus. The fight is ongoing; unsolved SARS-CoV-2 protein structures and emerging variants remain that require the continued attention and skills of the structural biology community.

A major factor at the base of the amazing work that has been accomplished so far was the prior research on other coronaviruses, such as SARS and MERS. Previous research empowered the remarkable response time and underscores the critical foundation of information that made it possible to mobilize in the face of the SARS-CoV-2 outbreak. We are therefore at a unique time not just to reflect on what has been done, but also to contemplate how the community can rapidly face new threats when they emerge. We find ourselves confronted by a set of compelling questions. What have we learned from the pandemic and the collective responses of scientific communities to this worldwide crisis? What features of the pipelines that we have been developing empower sharing and cross-fertilization across disciplines? What other health crises could benefit from the approaches that we have developed? We believe that this is a critical time for thinking about how to continue to build strategies for constructing frameworks for response to emerging threats.

The structural biology response to the COVID-19 pandemic has allowed the world to see the virus that has been attacking them. It has aided vaccine and therapeutic development and has shown foresight in the investment in structural capabilities, MX and CryoEM. With the intense focus on a single area in a short time period, it has nicely illustrated the complementary strengths of approaches. MX is used for the rapid study of smaller components and the high-resolution study of many potential therapeutic ligand complexes, while CryoEM has shown its strength in larger components. We do not doubt that the currently small overlap of methods illustrated in Fig. 3 ▸ will grow over time, as CryoEM continues to develop and new MX capabilities become available. We are well placed to fight the next pandemic, but will be even better placed in the future. Robotic approaches will improve, computational techniques will become more powerful and developments in the MX arena will allow studies at physiological temperatures. When combined, structural biology methods provide us with a complete picture to fight a virus; when techniques are used alone, important areas may be missed.
